# Transduction of Primary AML Cells with Lentiviral Vector for *In Vitro* Study or *In Vivo* Engraftment

**DOI:** 10.1016/j.xpro.2020.100163

**Published:** 2020-11-25

**Authors:** Aaron D. Schimmer, Rashim Pal Singh, Ayesh K. Seneviratne, Geethu E. Thomas, Neil MacLean, Rose Hurren

**Affiliations:** 1 Princess Margaret Cancer Centre, University Health Network, Room 8-706, 101 College St., Toronto, ON M5G1L7, USA

**Keywords:** Cancer, Stem Cells

## Abstract

We describe a method to silence genes in primary acute myeloid leukemia cells by transducing them with shRNA in lentiviral vectors. The transduction of primary non-adherent cells is particularly challenging. The protocol will aid in performing such experiments and is particularly helpful to prepare cells for *in vivo* engraftment studies. Use of a special medium supplemented with cytokines preserves the viability of the leukemic stem cells and their ability to engraft the marrow of immune-deficient mice.

For complete details on the use and execution of this protocol, please refer to Singh et al. (2020).

## Highlights

• An optimized protocol to silence genes in primary AML cells
• Transduces primary AML cells with shRNA in lentiviral vectors
• Preserves viability of the primary cells
• Suitable for downstream assays including cell viability and *in vivo* engraftment

## Graphical Abstract

**Figure:**
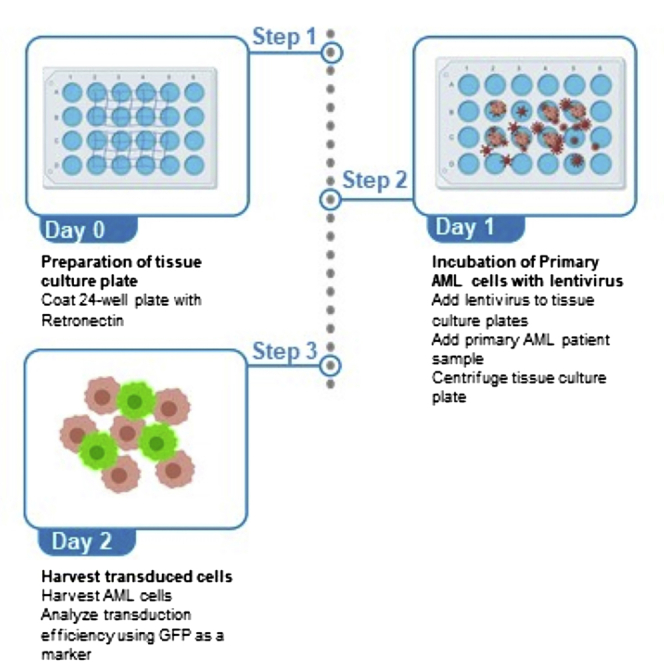

## Before You Begin

This protocol requires a concentrated stock of shRNA in lentivirus to infect the primary cells. A brief outline for the preparation of the concentrated virus is provided below. The reader is also referred to (Seneviratne et al., 2019) for more details. Proper institutional approvals for biosafety, use of animals, and human samples should be obtained before starting this protocol. This protocol provides a reliable transduction efficiency in hard-to-transduce primary AML cells with minimal toxicity. We have used this protocol to transduce 18 patient samples

### Preparation of Concentrated Lentiviral Stock to Infect Primary AML Cells

Preparation of concentrated lentivirus is necessary for the transduction of primary cells with lentiviral vectors. Concentrated virus is produced in 293T cells. Grow cells in DMEM + 10% heat inactivated FBS in a T175 flask up to 75% confluency.

**CRITICAL:** No antibiotics should be added to the medium.

**CRITICAL:** The following steps should be performed in a lab equipped to handle samples infected with lentivirus. Double gloves, lab coat, and protective gear should be worn during handling of the virus.

#### Day 1

1. Transfect the 293T cells with the lentiviral packaging plasmids containing the shRNA using transfection reagents such as TransIT-LT1 (Mirus Bio, #MIR2300).

#### Day 2

2. Discard the medium from the transfected 293T cells into 10% bleach and add 72 mL of Harvest Media (500 mL DMEM +10% heat inactivated FBS + 5 mL 100× Pen/Strep + 32 mL 20% BSA) per T175 flask.

#### Day 3

3. Transfer 35 mL of the supernatant medium containing viral particles to 50 mL conical tubes.
4. Centrifuge the medium at 1,200 rpm for 5 min to pellet any contaminating 293T cells.
5. Transfer the viral supernatant to fresh conical tubes.

**CRITICAL:** Avoid disturbing the small pellet while transferring the medium.

6. Add Lenti-X virus concentrator (Takara #631232) at ratio of 1 part Lenti-X to 3 parts medium (11.7 mL). Mix gently and incubate at 4°C overnight.

#### Day 4

7. Centrifuge viral supernatant at 1,500 × *g* for 45 min at 4°C to pellet the virus.
8. Remove the supernatant and re-suspend the pellet in HBSS/25 mM HEPES (2.5 mL per 50 mL tube). Aliquot and freeze at −75°C until further use.

**CRITICAL:** The virus pellet will be small and medium should be removed carefully. Repeat the centrifugation step if the pellet was disturbed during decanting the medium.

9. Check the virus concentration prior to freezing by Lenti-X GoStix Plus test strips (Takara #631281). The concentration is calculated by comparing the test band to the control band to ensure a viral concentration >5 × 10^5^ IFU/mL.

### Prepare Tissue Culture Plates (Day 0)

10. Coat 24-well non-tissue culture plate (Falcon #351147) with Retronectin by adding 250 μL of Retronectin-PBS stock solution (20 μg/mL). Incubate for 2 h at room temperature. Prepare 20 wells per plate and leave the corner wells untreated for the “No Virus” control samples.
11. Ensure Retronectin covers the entire bottom surface area of the wells. Shake and tap the plate or use a pipet to ensure even coverage.
12. Aspirate Retronectin from the wells (start from the edges of the well) and block Retronectin-coated wells with 500 μL 2% BSA PBS solution for 30 min at 20°C–22°C.
  a. To make 2% BSA PBS, add 2 g of BSA to 100 mL of sterile PBS.
  b. Mix using magnetic stirrer.
  c. Filter 2% BSA PBS mix using a PVDF 0.22 μM sterile filter. Store at 4°C.
13. Aspirate BSA solution from the wells, seal the edges of the plates with parafilm and store plates at 4°C. Plates can be store for up to 1 week.

**Pause Point:**

## Key Resources Table

**Table:** 

REAGENT or RESOURCE	SOURCE	IDENTIFIER
**Antibodies**		

Anti-human CD45-PE	BD Biosciences	555483
Anti-human CD33 –PE-Cy5	BD Biosciences	551377
Anti-mouse CD122	Lab Stock	N/A

**Biological Samples**		

Human AML Patient Samples	Leukemia Biobank, Princess Margaret Hospital	N/A

**Chemicals, Peptides, and Recombinant Proteins**		

Alpha MEM	Lab Stock	N/A
X-VIVO™ 10 Chemically Defined, Serum-free Hematopoietic Cell Medium	Lonza	Cat# 04-380Q
Dulbecco’s Modification Eagle’s Medium (DMEM)	Wisent Inc.	Cat# 319-005-CL
Fetal Bovine Serum	Sigma-Aldrich®	Cat# F1051
Recombinant Human IL-3 (8227, and Primary Sample Transductions)	PeproTech	Cat# 200-03
Recombinant Human SCF (78227, and Primary Sample Transductions)	PeproTech	Cat# 300-07
Recombinant Human Flt3-Ligand (8227, and Primary Sample Transductions)	PeproTech	Cat# 300-19
Recombinant Human TPO (8227, and Primary Sample Transductions)	PeproTech	Cat# 300-18
Recombinant Human IL-6 (8227, and Primary Sample Transductions)	PeproTech	Cat# 200-06
BIT 9500 Serum Substitute (8227, and Primary Sample Transductions)	STEMCELL Technologies	Cat# 09500
Granulocyte colony stimulating factor (G-CSF), [Filgrastim]	Amgen Canada	CAS#121181-53-1
Protamine Sulfate	MP Biomedicals	Cat#194729
Dimethyl sulfoxide (DMSO)	Sigma-Aldrich®	Cat# D8418
Bovine Serum Albumin	Sigma-Aldrich®	Cat# A9647
RetroNectin® Recombinant Human Fibronectin Fragment	Takara	Cat# T100A

**Critical Commercial Assays**		

Go-STIX test strips	Takara	Cat# 631281
Lenti-X™ Concentrator	Takara	Cat# 631231

**Experimental Models: Organisms/Strains**		

Mouse: NOD/SCID	Jackson Laboratory	Cat# 001303

## Step-By-Step Method Details

### Interaction of Primary AML Cells with Concentrated Virus (Day 1)

**Timing: 6–7 h**

In this section the virus will be spun down onto Retronectin-coated plates. The primary AML cells will be incubated with virus for transduction.

**CRITICAL:** Next steps should be performed in a containment lab equipped to handle viral samples.

1. Thaw the vials of concentrated virus in a 37°C water bath.
2. Add the 500 μL of concentrated virus onto Retronectin-coated plates (none in corner wells), spin plates at 3,000 rpm for 5 h at 4°C.

***Alternatives:*** This step can be performed at 20°C–22°C if preferred as no severe toxicity from the concentrated virus was observed using this temperature.

3. After spinning the above plates with concentrated virus for 5 h, pipette out 400 μL and leave 100 μL of virus in the wells.
4. During the 5-h spin, prepare the primary AML sample (frozen in 90% FBS +15 U/mL of heparin + 10% DMSO. As an alternative, samples may be frozen in alpha MEM + 5% FBS + 10% DMSO): Thaw X-VIVO 10 medium (Lonza, 04-380Q) and add to the medium BIT 9500 serum substitute at 4:1 ratio (Stem Cell Technologies #09500) along with cocktail of human cytokines and growth factors (GFs) IL3 (10 ng/mL), SCF (50 ng/mL), FLT3 ligand (50 ng/mL), G-CSF (10 ng/mL), TPO (25 ng/mL) and IL-6 (20 ng/mL).

**CRITICAL:** After the addition of cytokines, the medium should not be heated more than 20 min in a 37°C water bath. In addition, the medium is photosensitive and should be covered with aluminum foil at all times.

**Pause Point:** Medium can be kept for ~2 months at 4°C.

5. Thaw frozen primary AML patient sample in 37°C water bath (5–10 million cells).
6. Add thawed AML sample to 50 mL IMDM+10% FBS medium. Spin at 2,000 rpm for 10 min to pellet. Re-suspend pellet in 10 mL X-Vivo + BIT + GFs medium at a cell count of 5 × 10^5^ cells/mL.

**CRITICAL:** Thaw the patient samples as fast as possible and add sample immediately to the medium. Otherwise, the cells will clump, and the sample will be lost.

7. Add 10 μL protamine sulfate (final concentration 5 μg/mL) to 10 mL of cells in medium.
8. Add 1 mL of AML cells (5 × 10^5^/mL) to all 24 wells (4 corner wells are for No Virus controls) and then spin plate at 1,300 rpm for 10 min to increase the interaction between the cells and lentiviral particles. The spin time can be increased depending on experimental needs.
9. Incubate AML patient sample cells with the virus for ~24 h at 37°C in a 5% CO_2_ incubator.

**CRITICAL:** Throughout the procedure use sterile filter tips and individually wrapped plastic pipettes.

### Collection of Transduced Cells (Day 2)

**Timing: 1–2 h**

In this section cells will be collected and transduction efficiency will be measured by calculating the percentage of GFP positive cells. The cells will be injected into the sub-lethally irradiated immune compromised mice.

10. Collect the cells from each well and transfer them to sterile 50 mL tubes.

**CRITICAL:** The medium in each well should be pipetted up and down several times before the cells are transferred to the tubes. If this step is not performed, the majority of cells will be lost.

11. No virus control cells and virus treated cells should be collected in separate tubes.
12. Spin transduced cells at 2,000 rpm for 10 min. Decant the supernatant into 10% bleach.
13. Re-suspend AML cells in fresh X-Vivo10 + BIT + GFs medium at a final count of 1 × 10^6^ cells/mL, final volume depends on the total cell count.
14. Aliquot 1 mL of cells to a new 24-well plate. The user can set up the plate as desired. A suggestion is given in Figure 1.

**Figure 1:**
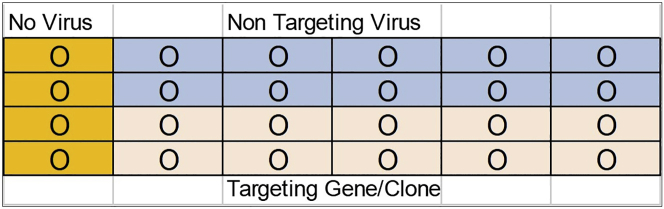
Plate Setup A suggested layout for the setup of the plate for the transduction of primary AML cells.
15. Leave overnight at 37°C, 5% CO_2_ incubator.
16. The percentage of GFP positive cells in total viable cell population (7AAD negative) can be measured in an aliquot of cells that were not injected into the mouse using a flow cytometer. These values are required to calculate engraftment potential at the end of in vivo experiments.

***Alternatives:*** GFP levels can be measured at days 3 and 4 as well.

17. To confirm the target gene knockdown, sort GFP+ cells at day 2–3 post transduction and perform RT-PCR.
18. Infected cells can be maintained in culture for up to 1 week for downstream *in- vitro* experiments.
19. To engraft the marrow of immune deficient mice, inject 3 × 10^5^ to 1 × 10^6^ of AML cells into immune deficient mice. One possible mouse strain are sub-lethally irradiated (200 rad) immunodeficient NOD/SCID (6–10 weeks) mice, conditioned with intraperitoneal injection of anti-mouse CD122 antibody generated in house using the TM-beta1 Hybridoma. (200 μg/mouse) 24 h prior to injection of cells. The engraftment was evaluated using anti-human CD45 and CD33.

## Expected Outcomes

**Figure 2:**
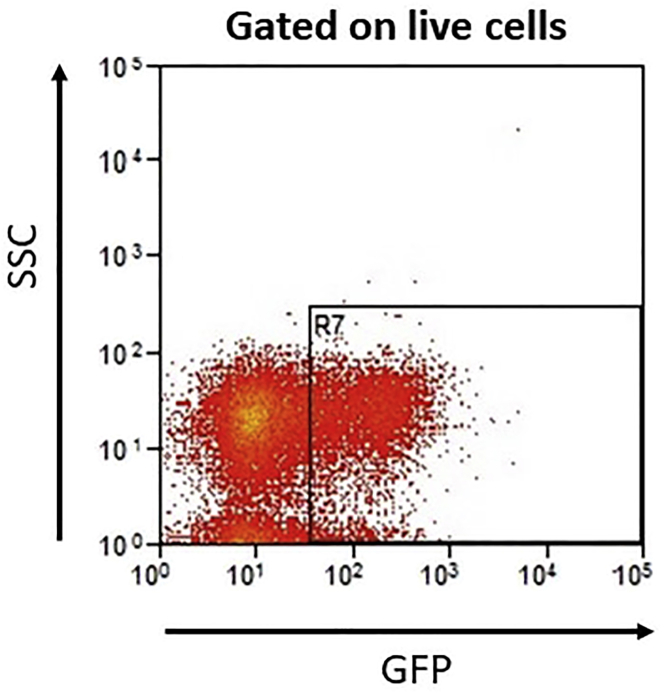
Transduction Efficiency *In Vitro* The percentage of GFP positive cells as measured by flow cytometry after transduction of primary AML cells with a lentiviral vector containing a GFP marker. A representative experiment is shown.

**Figure 3:**
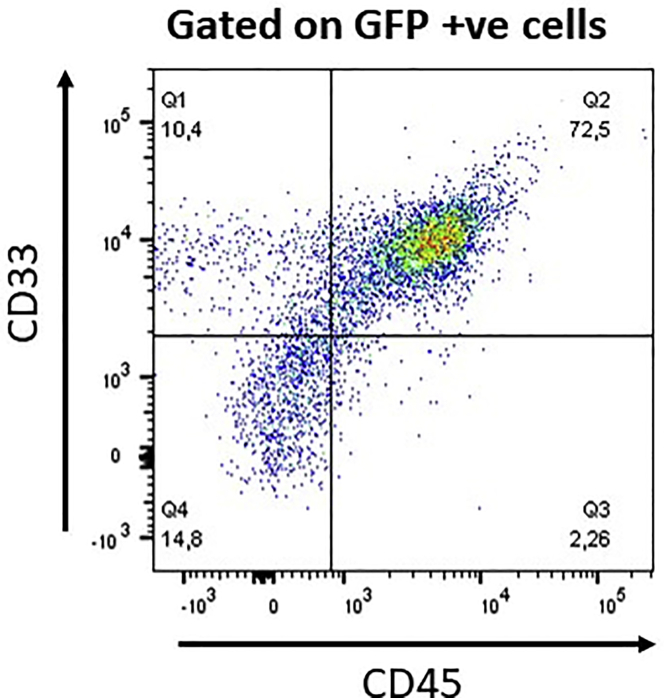
Identification of Transduced Cells *In Vivo* Flow cytometric analysis of transduced primary AML cells isolated from the femur of a mouse.

The above protocol will generate primary AML blasts with target gene knockdown in infected cells while preserving their stem cell properties. The transduction efficiency will be between 15% and 30% (Figure 2 ,Table 1). After transduction, mRNA target knockdown and changes in gene expression in sorted cells can be assessed by qRT-PCR. By using flow cytometry and gating on transduced cells, functional studies including cell viability, and engraftment into immune deficient mice in primary and secondary transplants can be measured (Figure 3).

**Table 1:** Transduction Efficiency in Primary AML Samples

Primary AML	1	2	3	4	5	6	7
Transduction efficiency (%)	22	12	26	27	27	18.9	25

## Limitations

The average transduction efficiency is between 15% and 30% as measured by GFP positive cells. These values must be kept in mind for downstream assays. The proliferation of primary AML cells in culture is limited.

## Troubleshooting

### Problem

Lower than expected transduction efficiency.

### Potential Solution

The transduction efficiency can be increased by enhancing the interaction of AML cells and lentiviral particles, by one or all of the following steps:

While coating the plates with Retronectin, make sure that the wells are evenly coated and the whole surface of the well is covered. While removing the Retronectin and blocking BSA solution, make sure wells are not scratched and the liquids are aspirated from the edges of the well.
The centrifugation of viral particles should be performed for a full 5 h, lower times may decrease the cell and virus interaction. Cells cannot be co-spun for such long duration as it will create viability issues.
Ensure that the X-Vivo + BIT + GFs medium has been prepared and stored properly as the medium preserves the viability of the AML cells. Over time, or with improper handling, the medium can spoil. After thawing of the medium and addition of cytokines, the medium is stable at 4°C for ~2 months. The medium is sensitive to heat and light. Prior to use it should not be warmed in a 37°C water bath for more than 20 min and tubes should always be covered with aluminum foil.

### Problem

Clumping of primary AML cells during thawing.

### Potential Solution

Always thaw the primary AML cells in a 37°C water bath. Work fast and as soon as the sample thaws transfer it to 50 mL of IMDM + 20% FCS. Always use 50 mL of medium, using lower volumes may increase clumping of cells. The number of cells thawed is potential issue.

### Problem

Non-Engraftment of GFP+ cells.

### Potential Solution

The handling of primary patient samples is very important. Repeated thawing and freezing could lead to depletion of stem cell population. In addition, the recipient mice should be of 6–10 weeks of age and should be kept in specific pathogen-free cages.

## Resource Availability

### Lead Contact

Further information and requests for resources and reagents should be directed to and will be fulfilled by the Lead Contact: Aaron D Schimmer (aaron.schimmer@uhn.ca).

### Materials Availability

This study did not generate any unique reagents

### Data and Code Availability

This study did not generate/analyze datasets of code.
